# *Piper betle* shows antioxidant activities, inhibits MCF-7 cell proliferation and increases activities of catalase and superoxide dismutase

**DOI:** 10.1186/1472-6882-12-220

**Published:** 2012-11-15

**Authors:** Noor Nazirahanie Abrahim, M S Kanthimathi, Azlina Abdul-Aziz

**Affiliations:** 1 Department of Molecular Medicine, Faculty of Medicine, University of Malaya, 50603 Kuala Lumpur, Malaysia; 2 University of Malaya Centre for Proteomics Research, University of Malaya, 50603 Kuala Lumpur, Malaysia

**Keywords:** *Piper betle*, Antioxidant, Phenolic, MCF-7, Cytotoxicity, Catalase, Superoxide dismutase, HPLC

## Abstract

**Background:**

Breast cancer is the most common form of cancer and the focus on finding chemotherapeutic agents have recently shifted to natural products. *Piper betle* is a medicinal plant with various biological activities. However, not much data is available on the anti-cancer effects of *P. betle* on breast cancer. Due to the current interest in the potential effects of antioxidants from natural products in breast cancer treatment, we investigated the antioxidant activities of the leaves of *P. betle* and its inhibitory effect on the proliferation of the breast cancer cell line, MCF-7.

**Methods:**

The leaves of *P. betle* were extracted with solvents of varying polarities (water, methanol, ethyl acetate and hexane) and their phenolic and flavonoid content were determined using colorimetric assays. Phenolic composition was characterized using HPLC. Antioxidant activities were measured using FRAP, DPPH, superoxide anion, nitric oxide and hyroxyl radical scavenging assays. Biological activities of the extracts were analysed using MTT assay and antioxidant enzyme (catalase, superoxide dismutase, glutathione peroxidase) assays in MCF-7 cells.

**Results:**

Overall, the ethyl acetate extract showed the highest ferric reducing activity and radical scavenging activities against DPPH, superoxide anion and nitric oxide radicals. This extract also contained the highest phenolic content implying the potential contribution of phenolics towards the antioxidant activities. HPLC analyses revealed the presence of catechin, morin and quercetin in the leaves. The ethyl acetate extract also showed the highest inhibitory effect against the proliferation of MCF-7 cells (IC_50_=65 μg/ml). Treatment of MCF-7 cells with the plant extract increased activities of catalase and superoxide dismutase.

**Conclusions:**

Ethyl acetate is the optimal solvent for the extraction of compounds with antioxidant and anti-proliferative activities. The increased activities of catalase and superoxide dismutase in the treated cells could alter the antioxidant defense system, potentially contributing towards the anti-proliferative effect. There is great potential for the ethyl acetate extract of *P. betle* leaf as a source of natural antioxidants and to be developed as therapeutics in cancer treatment.

## Background

Breast cancer is the third most common cause of cancer deaths worldwide and is the most common form of cancer in women. The cause of breast cancer is multi-factorial, with hormonal, genetic and environmental factors playing a role in its pathogenesis. The current treatment strategies involve chemotheraphy, radiation theraphy, hormones and surgery. More recently, the focus on finding chemotherapeutic agents have shifted towards natural products. Various plants and their bioactive compounds have been shown to have anti-carcinogenic and anti-proliferative effects towards breast cancer cells [1,2]. Studies have also reported positive correlation between antioxidant activities of plants and their anti-proliferative effects, suggesting the potential action of antioxidants in inhibiting cancer cell growth [3]. Phenolic compounds such as epigallocatechin gallate and quercetin, which have high antioxidant activities, demonstrated anti-proliferative effects against breast cancer cell lines [1,2]. This has prompted further studies investigating the possible involvement of antioxidant-rich plants as anticancer agents.

*Piper betle* is a medicinal plant that is traditionally used in catarrhal and pulmonary affections, as a digestive and carminative and as a stimulant of pancreatic lipase [4-6]. *P. betle* belongs to the Piperaceae family and is thought to originate from South East Asia. The leaves of the plant are commonly chewed with areca nut. Scientifically, studies have reported the biological benefits of *P. betle* to include inhibition of platelet aggregation [7], anti-diabetic activities [8], immunomodulatory properties [9] and anti-allergic activities [10]. Some of these observed biological activities were attributed to the high antioxidant activities of this plant [11,12]. Several studies have been conducted on the effect of *P. betle* in reducing various types of tumors. The aqueous extract of *P. betle* prevented formation of tumors when fed to rats in the initiation phase of induced-mammary carcinogenesis but could not inhibit tumor growth when fed to rats with induced mammary carcinogenesis [13]. Furthermore, the leaves of *P. betle* has strong anti-tumor promoting activities in Raji cells [14] whereas the aqueous extract was reported to show anti-proliferative action towards kB cells, indicating their potential in treating oral cancer [15]. Not much data is available on the anti-proliferative effects of *P. betle* on breast cancer. Since this plant contains high antioxidant activities, it can potentially exhibit anti-proliferative effects. Due to the current interest in the potential effects of antioxidants from natural products in breast cancer treatment, we attempted to investigate the antioxidant activities and cytotoxic effect of the leaves of *P. betle* against the breast cancer cell line, MCF-7. Studies have reported the antioxidant activities of *P. betle* leaf in aqueous extracts [8,11,16]. In this study, we report the effect of solvents of varying polarities on the antioxidant activities of the leaves of *P. betle.* We also investigated the anti-proliferative and antioxidant status of the various plant extracts on the breast cancer cell line, MCF-7.

## Methods

### Materials

Solvents used for extraction of plants were purchased from Fisher Scientific. High performance liquid chromatography (HPLC) grade phenolic standards, gallic acid, quercetin and rutin were obtained from Sigma Chemical Co. (St. Louis, USA). All the standards had purities above 95%. HPLC grade acetonitrile and other analytical grade chemicals and reagents were obtained from the general suppliers.Water used was of Millipore quality.

### Sample preparation

The leaves of *Piper betle* were cleaned of any dirt and rinsed with water. The leaves were left to air-dry and subsequently ground into powder and was kept at −20°C for further analyses. The dried powder was extracted through sequential extraction using hexane, ethyl acetate, methanol and water. Briefly, 10 g of the powder was mixed with 100 ml of hexane and was left to stir on a hot plate at a temperature of 40°C. The extract was filtered after 6 h and the resulting residue was re-extracted twice with the same solvent. The extraction process was continued with the remaining residue using solvents of increasing polarity, with each extraction performed three times using the same solvent. The hexane, ethyl acetate and methanol extracts from the three extractions were pooled and evaporated using the rotary evaporator. The water extract was lyophilized on the freeze-drier. The extract was subsequently dissolved in 10% dimethylsulphoxide (DMSO) and kept at −20°C until further analyses.

### Analysis of phenolic content

The phenolic content of the extracts was determined through the Folin-Ciocalteu assay developed by Singleton & Rossi (1965) [17]. Phenolic compounds, at basic pH, are capable of reducing the phosphomolybdic and phosphotungstic heteropoly acid reagent, forming a blue complex which can be measured at a wavelength of 765 nm. Briefly, 10 μl of extract was mixed with 0.5 ml of Folin-Ciocalteu reagent, incubated for 5 min, followed by the addition of 0.35 ml sodium carbonate (0.115 mg/ml). The mixture was allowed to stand in the dark for 2 h before absorbance readings were taken at 765 nm. Gallic acid was used as standard and was analysed as above. The concentration of phenolics in the extracts was expressed as miligram gallic acid equivalents per g dried weight (mg GAE/g dried weight). All analyses were carried out in triplicate.

### Analysis of flavonoid content

Analysis of flavonoid content was done using the aluminium chloride colorimetric assay. The assay is based on the formation of an acid-stable complex of aluminium chloride with the C-4 keto group and either the C-3 or C-5 hydroxyl group of flavonoids [18]. Briefly, 500 μl of plant extract was mixed with 1.5 ml of ethanol (95%), 0.1 ml of 10% aluminium chloride, 0.1 ml of 1 M sodium acetate and 2.8 ml distilled water. Absorbance of the yellow-green complex was measured at 415 nm after 30 min. Quercetin was used as standard and analysed as above. Flavonoid content of the plant extracts was expressed as mg quercetin equivalents per gram dried weight (mg QE/g dried weight). Each sample was analysed in triplicates.

### Ferric reducing activity

The ferric reducing activity of the plant extracts was estimated based on the ferric reducing ability of plasma (FRAP) assay developed by Benzie & Strain (1996) [19]. This assay measures the ability of antioxidants in the samples to reduce the ferric to a colored ferrous product at 593 nm. Reagents for this assay consisted of 300 mmol/L acetate buffer, 10 mmol/L 2,4,6-tripyridyl-s-triazine (TPTZ) in 40 mmol/L of HCl and 20 mmol/L FeCl_3_.6H_2_O. The working reagent was prepared fresh by mixing 25 mL acetate buffer with 2.5 mL TPTZ solution and 2.5 mL FeCl_3_.6H_2_O. In the assay, 900 μl of the working reagent was mixed with 30 μl of sample and 90 μl of water. Absorbance of the mixture was read at 593 nm every 15 s for four minutes. Quercetin and rutin were used as positive controls and analysed in parallel. All experiments were performed in triplicate. A standard curve of FeSO_4_.7H_2_O (0 – 1000 μmole/L) was plotted for determination of the ferric reducing activity. Results were expressed as millimole per gram of dried weight.

### DPPH radical scavenging activity

This assay was used to evaluate the radical scavenging activity of antioxidants in the plant extracts against a chemically-synthesised radical, 2,2-diphenyl-1-picryl-hydrazyl (DPPH). In this assay, 100 μl of the extract (0–400 μg/ml) was added to 600 μl of DPPH reagent (100 μM), mixed thoroughly and incubated in the dark at room temperature for 20 min. The decrease in absorbance was measured at 517 nm. The experiment was carried out in triplicate using Trolox as standard. Quercetin and rutin were used as positive controls. The DPPH radical scavenging activity was calculated using the following equation:

(1) $$\%\;\text{of inhibition}=\left[{\text{Absorbance}}_{\left(\text{blank}\right)}-{\text{Absorbance}}_{\left(\text{sample}\right)}/{\text{Absorbance}}_{\left(\text{blank}\right)}\right]\text{X}\;100$$

Results were expressed as IC_50_, i.e. concentration of the plant extract required to inhibit 50% of the DPPH radicals.

### Superoxide anion radical scavenging activity

The superoxide anion radical scavenging activity was measured based on the method by Siddhuraju & Becker (2007) [20]. Superoxide anion reacts with nitroblue tetrazolium (NBT) to generate a colored compound which can be measured at a wavelength of 560 nm. The reaction mixture contained 0.1 M phosphate buffer (pH 7.4), 150 μM NBT, 60 μM phenazine methosulphate, 468 μM NADH and the plant extracts (0–500 μg/ml), added in that sequence. Absorbance of the mixture was read at 560 nm after a 10 min incubation period in the dark. Trolox was used as standard and quercetin and rutin were used as positive controls. All analyses were done in triplicates. Superoxide anion scavenging activity was calculated using the following equation:

(2) $$\%\;\text{of inhibition}=\left[{\text{Absorbance}}_{\left(\text{blank}\right)}-{\text{Absorbance}}_{\left(\text{sample}\right)}/{\text{Absorbance}}_{\left(\text{blank}\right)}\right]\;\text{X}\;100$$

Results were expressed as IC_50_, i.e. concentration of the plant extract required to inhibit 50% of the superoxide anion radicals.

### Nitric oxide radical scavenging activity

The nitric oxide radical scavenging activity was conducted based on the method of Rai, Wahile, Mukherjee, Saha, & Mukherjee (2006) [21]. Briefly, 0.5 ml of sodium nitroprusside (10 mM) was mixed with 0.5 ml of the plant extract (0–500 μg/ml). The mixture was kept in the dark at room temperature for 150 min. Thereafter, 1 ml of sulfanilic acid reagent was added to 0.5 ml of the reaction mixture and incubated for 5 min. One ml of 0.1% naphthyl ethylene diamine dihydrochloride was then added, mixed and incubated for 30 min at 25°C before the absorbance was read at 540 nm. Trolox was used as standard while quercetin and rutin were used as positive controls. All analyses were done in triplicate. Nitric oxide scavenging activity was calculated using the following equation:

(3) $$\%\;\text{of inhibition}=\left[{\text{Absorbance}}_{\left({}_{\text{blank}}\right)}-{\text{Absorbance}}_{\left({}_{\text{sample}}\right)}/{\text{Absorbance}}_{\left({}_{\text{blank}}\right)}\right]\text{X}\;100$$

Results were expressed as IC_50_, i.e. concentration of the plant extract required to inhibit 50% of the nitric oxide radicals.

### Hydroxyl radical scavenging activity

This assay measures the competition between deoxyribose and antioxidants in the plant extract for hydroxyl radicals generated from the Fe^3+^/ascorbate/H_2_O_2_ system. Briefly, 0.2 ml of plant extract was mixed with 0.2 ml of ferric chloride (100 mM), 0.2 ml of hydrogen peroxide (1.25 mM), 0.2 ml of deoxyribose (2.5 mM) and 0.2 ml of ascorbic acid (100 mM). The reaction mixture was then incubated for 1 h at 37°C before the addition of 1 ml of thiobarbituric acid solution and 1 ml of trichloroacetic acid. The mixture was heated for 30 min at 80°C and cooled on ice. Absorbance of the mixture was measured spectrophotometrically at 532 nm. Each analysis was done in triplicates. Hydroxyl radical scavenging activity was calculated using the following equation:

(4) $$\%\;\text{of inhibition}=\left[{\text{Absorbance}}_{\left({}_{\text{blank}}\right)}-{\text{Absorbance}}_{\left({}_{\text{sample}}\right)}/{\text{Absorbance}}_{\left({}_{\text{blank}}\right)}\right]\text{X}\;100$$

Results were expressed as IC_50_, i.e. concentration of the plant extract required to inhibit 50% of the hydroxyl radicals.

### Analyses of phenolic compounds using high performance liquid chromatography (HPLC)

The dried powder of *P. betle* leaf was subjected to acid hydrolysis to release free polyphenols from their glycosides, following the procedure described in Razali, Mat-Junit, Abdul-Muthalib, Subramaniam, & Abdul-Aziz (2012) [22]. Briefly, In a glass vial, 20 mg of the dried *P.betle* powder in 1.2 M HCl and 50% methanol was heated at 90°C for 2 h. The mixture was left to cool and was centrifuged at 3000 *g* for 10 min. The supernatant was collected and kept at −20°C for the HPLC analysis.

The samples were analysed using a Shimadzu HPLC system. Reverse phase separation was performed at 30°C using a Waters C_18_ column (3.9 X 150 mm) (Waters, USA). The mobile phase consisted of trifluoroacetic acid (TFA) in water at pH 2.6 (solvent A) and acetonitrile (solvent B). The flow rate was kept at 1 ml/min and the gradient programme consisted of: 7% to 40% B for 20 min, 40% to 100% B for 6 min and 100% to 7% B for 9 min. The eluted peaks were monitored at 260 nm. Data acquisition and processing was performed using a Lab Solution chromatography manager. 200 μl of sample was injected into the HPLC. All samples were analyzed in triplicate.

### Cell culture

MCF-7 human breast cancer cells were utilised for the anti-proliferation study. The cells were maintained in RPMI 1640 culture medium supplemented with 10% (v/v) foetal bovine serum (FBS) (Flowlab, Australia), 10 μg/ml bovine serum albumin and antibiotics and kept at 37°C in T-25 tissue culture flasks (TPP, Switzerland). Cell were grown to confluence in a humidified atmosphere containing 5% CO_2_.

### Cell viability using MTT assay

MCF-7 cells were seeded in 96-well culture plates (5 X 10^3^ cells/well) and allowed to attach for 24 h before treatment. Varying concentrations of the extracts of *P. betle* (25–200 μg/ml), dissolved in DMSO were added to each well. The final concentration of DMSO in the treatment wells was less than 1%. The plates were incubated at 37°C for 48 h.

Cell viability following treatment with the *P. betle* extracts was measured using the 3-(4,5-dimethylthiazol-2-yl)-2,5-diphenyltetrazolium bromide (MTT) assay. MTT is reduced by mitochondrial succinate dehydrogenase in viable cells, forming purple formazan crystals that can be measured at 595 nm. Briefly, 5 mg/ml MTT reagent in PBS were added to each treatment well and were incubated for 4 h. The media containing the MTT reagent were subsequently removed and replaced with 100 μl of acidic isopropanol. The resulting absorbance was measured at 595 nm and cell viability was calculated based on the formula:

(5) $$\text{Percentage of inhibition}\;\left(\%\right)=\left[\left(\text{total cells}–\text{viable cells}\right)/\text{total cells}\right]\text{X}\;100$$

All analyses were performed in triplicate.

### Analyses of antioxidant enzyme activities

MCF-7 cells (1 x 10^6^) were seeded in T-25 flasks containing RPMI 1640 medium supplemented with 10% FBS and allowed to attach for 24 h before treatment. Cells were treated with the ethyl acetate extract of *P.betle* at a final concentration of 64 μg/ml (IC_50_ concentration determined from the MTT assay), at varying time points (0, 24 and 48 h). Following incubation, the cells were washed with PBS and detached using a scraper. Cells were then lysed in 1 ml of cold PBS using a sonicator, centrifuged for 10 min at 10000 rpm at 4°C and the resulting supernatant was used for the antioxidant enzyme assays.

### Catalase (CAT) assay

This assay was performed using the Catalase Assay Kit from Cayman Chemicals (USA). The assay is based on the reaction of CAT with methanol in the presence of hydrogen peroxide, producing formaldehyde which is measured colorimetrically using 4-amino-3-hydrazino-5-mercapto-1,2,4-triazole (Purpald) as the chromogen. Purpald forms a bicyclic heterocycle with aldehydes, which upon oxidation changes from colorless to a purple color. The assay was performed according to the manufacturer’s instructions. CAT activity in each sample was expressed in nmol/min/ml using the following equation:

(6) $$\text{CAT activity}=\left(\text{μM of sample}/20\;\text{min}\right)\;\text{X sample dilution}$$

One unit is defined as the amount of enzyme that caused the formation of 1.0 nmol of formaldehyde per minute at 25°C.

### Superoxide dismutase (SOD) assay

This assay was performed using the superoxide dismutase Assay Kit from Cayman Chemicals (USA). The assay uses tetrazolium salt for detection of SOD generated by xanthine oxidase. The assay was performed according to the manufacturer’s instructions. SOD activity was calculated using the following equation:

(7) $$\text{SOD activity}\;\left(\text{U}/\text{ml}\right)=\left\{\left[\left(\text{sampleLR}–\text{y}-\text{intercept}\right)/\text{Slope}\right]\text{X}\;\left(0.23\;\text{ml/}0.01\;\text{ml}\right)\right\}\text{X sample dilution}$$

One unit is defined as the amount of enzyme needed to catalyze 50% dismutation of the superoxide radical.

### Glutathione peroxidase (GPx) assay

This assay was performed using the glutathione peroxidase Assay Kit from Cayman Chemicals (USA). This experiment measures GPx activity through a coupled reaction with glutathione reductase. The assay was performed according to the manufacturer’s instructions. GPx activity was calculated using two formulae:

(8) $$\Delta {\text{A}}_{340}/\text{min}=\left[{\text{A}}_{340}\left(\text{Time}\;2\right)-{\text{A}}_{340}\left(\text{Time}\;1\right)\right]/\left[\text{Time}\;2\;\left(\text{min}\right)-\text{Time}\;1\;\left(\text{min}\right)\right]$$

Time 1 represents absorbance at 0 min, Time 2 is the absorbance at 5 min and ∆A_340_/min. refers to the change in absorbance per minute obtained from the standard curve. Thus, activity of GPx in the samples was determined as:

(9) $$\text{GPx activity}\left(\text{nmol}/\text{min}/\text{ml}\right)=\left(\Delta {\text{A}}_{340}\min^{-1}/0.00373\;{\text{μM}}^{-1}\right)\text{X}\;\left(0.19\;\text{ml/}0.02\;\text{ml}\right)\;\text{X sample dilution}$$

One unit is defined as the amount of enzyme that caused the oxidation of 1.0 nmol of NADPH to NADP^+^ per minute at 25°C.

### Statistical analyses

All analyses were done in triplicate. All data were analyzed using the SPSS statistical software for Windows, version 17.0. An independent t-test was used for comparison of means between groups. Pearson correlation coefficient was used to determine the relationship between phenolic content in the plant extracts with the respective antioxidant activities. Differences between means at the 95% (p<0.05) confidence level were considered statistically significant.

## Results

### Phenolic and flavonoid content

The ethyl acetate extract of *P. betle* had the highest phenolic content, more than 3 fold and 16 fold higher than the hexane and methanol extracts, respectively whereas the aqueous extract had the lowest amount (Table 1). In contrast, the ethyl acetate extract had the lowest amount of flavonoids whereas the methanol extract had the highest flavonoid content.

**Table 1:** **Phenolic content, flavonoid content and antioxidant activities of the extracts of *P. betle***

**Extracts**	**Phenolic content**	**Flavonoid content**	**Ferric reducing**	**DPPH radical scavenging**	**Superoxide anion radical scavenging**	**Nitric oxide radical scavenging**	**Hydroxyl radical scavenging**
	**(mg GAE/g dried weight)**	**(mg QE/g dried weight)**	**(mmol/g dw)**	**(IC**_**50**_**)**	**(IC**_**50**_**)**	**(IC**_**50**_**)**	**(IC**_**50**_**)**
Aqueous	47.72 ± 5.38^a^	13.39 ± 1.12^a^	0.347 ± 0.01^a^	nd	79.3 ± 1.15^a^	57.7 ± 2.52^b,c^	313.3 ± 32.15^a^
Methanol	52.25 ± 5.49^a^	19.85 ± 0.10^b^	0.476 ± 0.01^a^	345.7 ± 4.04^a^	288.3 ± 2.89^b^	143.3 ± 20.21^a^	nd
Ethyl acetate	852.3 ± 4.71^b^	7.39 ± 1.57^c^	6.052 ± 0.10^b^	40 ± 0.00^b^	48.3 ± 4.73^c^	52.3 ± 6.66^b,c^	416.7 ± 15.27^a^
Hexane	266.92 ± 6.06^c^	10.47 ± 0.63^d^	0.904 ± 0.01^c^	144.3 ± 1.15^c^	nd	94.3 ± 22.28^a,b^	nd
Quercetin	-	-	6.174 ± 0.09^b^	30.0 ± 0.00^d^	40.0 ± 0.00^c^	71.7 ± 2.89^a,b,c^	153.0 ± 110.48^b^
Rutin	-	-	2.279 ± 0.07^d^	33.7 ± 1.15^d^	44.0 ± 5.29^c^	81.3 ± 1.15^a,b,c^	nd

Results were expressed as means ± std. dev. (n=3).

IC_50_ is defined as concentration of plant extracts that inhibited 50% of the radicals.

Values with different superscript letters within the same column are significantly different (p < 0.05).

nd = not detected.

### Ferric reducing activity

The ferric reducing activities of the plant extracts in descending order are ethyl acetate > hexane > methanol > aqueous extracts (Table 1). The ferric reducing activities of the hexane, methanol and aqueous extracts were less than 1 mmol/g dried weight whereas the ethyl acetate extract had reducing activity above 6 mmol/g dried weight. The ferric reducing activity of the ethyl acetate extract almost matched that of the positive control quercetin and was three fold higher than the positive control rutin, implying its potent activity.

### DPPH radical scavenging activity

Similar to the ferric reducing assay, DPPH radical scavenging activity was highest in the ethyl acetate extract, followed by the hexane, methanol and aqueous extracts (Table 1). The ethyl acetate extract had an IC_50_ value of 40 μg/ml, slightly higher than quercetin (IC_50_=30 μg/ml) and rutin (IC_50_=33.7 μg/ml). The IC_50_ values of the hexane and methanol extracts were more than 100 μg/ml whereas the aqueous extract did not reach 50% inhbition of the DPPH radicals at the concentration tested, indicating their weakness as DPPH radical scavengers.

### Superoxide anion radical scavenging activity

The ethyl acetate extract demonstrated the highest superoxide anion radical scavenging activity (IC_50_=48.3 μg/ml) followed in descending order by the aqueous and the methanol extracts whereas the hexane extract did not reach 50% inhibition of the radicals at the highest concentration used in this study (500 μg/ml). The IC_50_ of the ethyl acetate extract was only slightly higher than quercetin and rutin (Table 1).

### Nitric oxide radical scavenging activity

Nitric oxide radical scavenging capacities of the extracts was highest in the ethyl acetate extract and this was followed by the aqueous > hexane > methanol extracts (Table 1). The ethyl acetate extract had an IC_50_ of 52.3 μg/ml, more potent than quercetin (IC_50_=71.7 μg/ml) and rutin (IC_50_=81.3 μg/ml). Interestingly, the aqueous extract was also reasonably effective with an IC_50_ of 57.7 μg/ml.

### Hydroxyl radical scavenging activity

The aqueous extract had the highest hydroxyl radical scavenging activity with an IC_50_ of 313.3 μg/ml, followed by the ethyl acetate extract (IC_50_=416.7 μg/ml) whereas the methanol and hexane extracts did not reach 50% inhibition of the hydroxyl radicals at the concentration used in the study (Table 1). The hydroxyl radical scavenging activities of the extracts were lower than quercetin (IC_50_=153.0 μg/ml).

### Correlation analyses

Pearson correlation analyses were performed to assess the relationship between the phenolic content of the plant extracts and the respective antioxidant activities (Table 2). A strong positive correlation was observed between phenolic content and ferric reducing activities (R^2^=0.982), followed by nitric oxide (R^2^=0.928) and DPPH (R^2^=0.923) radical scavenging activities. In addition, a moderate positive correlation was seen between phenolic content and superoxide anion (R^2^=0.628) radical scavenging activities whereas there was no significant correlation between phenolic content and hydroxyl radical scavenging activities.

**Table 2:** **Pearson correlation analyses of the phenolic content and antioxidant activities of the extracts of *P. betle***

	**FRAP**	**DPPH**	**Superoxide anion**	**Nitric oxide**	**Hydroxyl radical**
Phenolic content	0.982^a^	0.923^a^	0.628^b^	0.928^a^	0.326

FRAP, ferric reducing antioxidant power; DPPH, 1,1-diphenyl-2-picryl hydrazyl radical-scavenging activity.

^a^ Correlation is significant at the 0.01 level.

^b^ Correlation is significant at the 0.05 level.

### Analyses of phenolic compounds using high performance liquid chromatography (HPLC)

**Figure 1:**
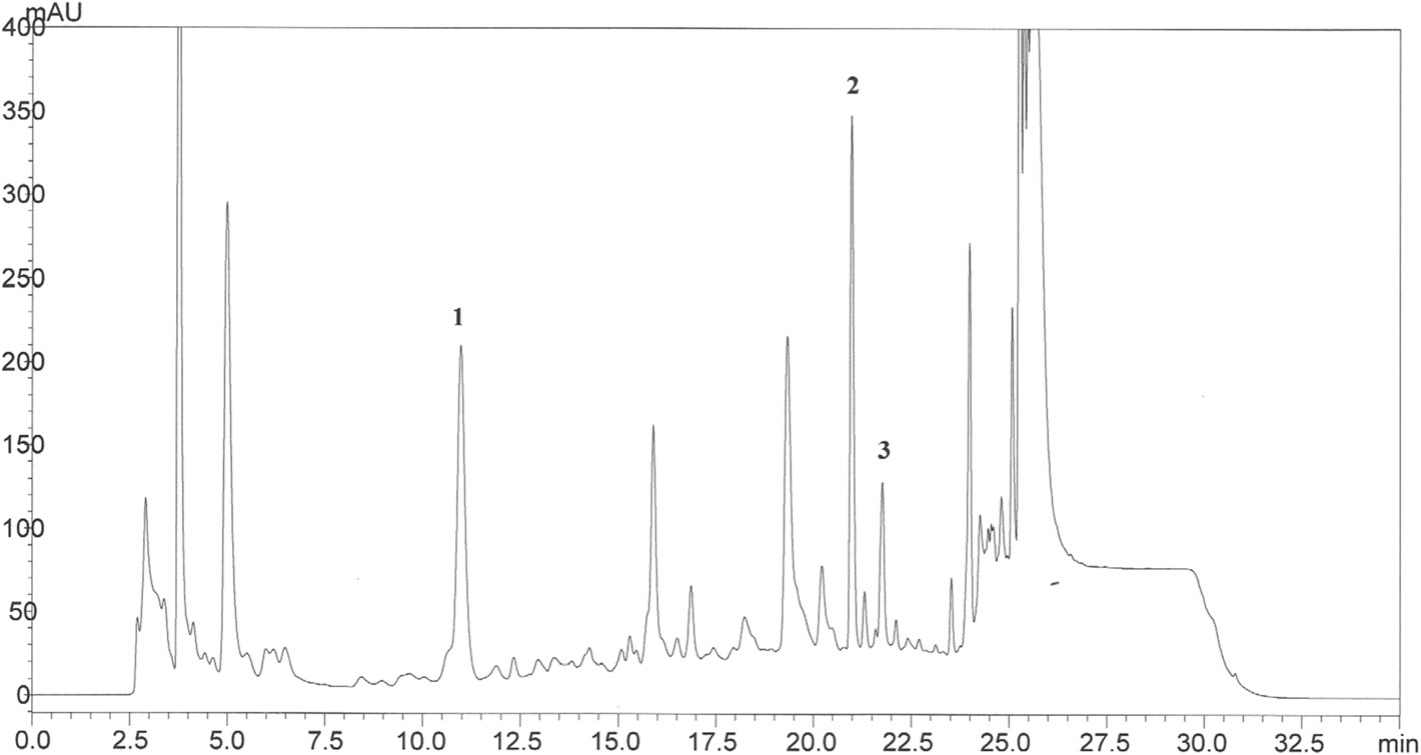
**HPLC chromatogram of the leaves of *P. betle *.** Reverse phase separation was performed using a C_18_ Waters column (3.9 X 150 mm). The mobile phase consisted of trifluoroacetic acid (TFA) in water at pH 2.6 (solvent A) and acetonitrile (solvent B). The flow rate was kept at 1 ml/min and the gradient programme consisted of: 7% to 40% B for 20 min, 40% to 100% B for 6 min and 100% to 7% B for 9 min. The eluted peaks were monitored at 260 nm. 200 μl of sample was injected into the HPLC. 1: catechin; 2: morin; 3: quercetin.

Figure 1 shows the HPLC traces of the leaves of *P.betle*. Catechin, morin and quercetin were detected in the samples. Phenolic compounds in the plant sample were detected by comparing the retention times of the peaks with the standards. Several peaks that did not correspond to the standards used in the HPLC analysis were observed in the chromatogram of the plant extract, between retention times 15–25 min.

### Cytotoxicity study using MCF-7 breast cancer cells

**Figure 2:**
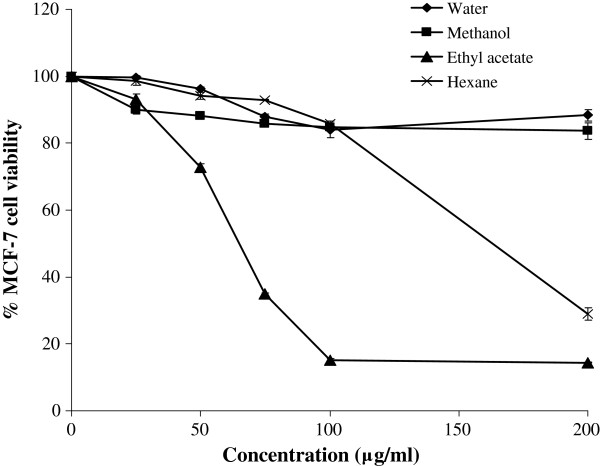
**The effects of the leaf extracts of *P. betle *on the proliferation of MCF-7 cells.** Cells were grown in RPMI 1640 medium supplemented with 10% (v/v) FBS, 10 μg/ml BSA and antibiotics, at 37°C in a humidified atmosphere containing 5% CO_2_. Confluent cells (5 X 10^3^ cells/well) were treated with the extracts of *P. betle* (25–200 μg/ml) for 48 h and cell viability was determined using the MTT assay.

To evaluate the cytotoxicity of the plant extracts, breast cancer cells (MCF-7) were treated with varying concentrations of the extracts and the MTT assay was performed. Amongst the four extracts, only the ethyl acetate and hexane extracts showed a dose-dependent inhibitory effects on the MCF-7 cells with IC_50_ values of 65.00 ± 0.00 and 163.30 ± 2.89 μg/ml, respectively (Figure 2). The ethyl acetate extract reached maximal growth inhibition of the MCF-7 cells at a concentration of 100 μg/ml. On the other hand, the water and methanol extracts did not exhibit cytotoxic activities, inhibiting less than 20% of the cells at the highest concentration tested (200 μg/ml). Due to the potent cytotoxic properties of the ethyl acetate extract of *P. betle*, subsequent analyses were conducted only on this extract.

### Catalase activity

**Figure 3:**
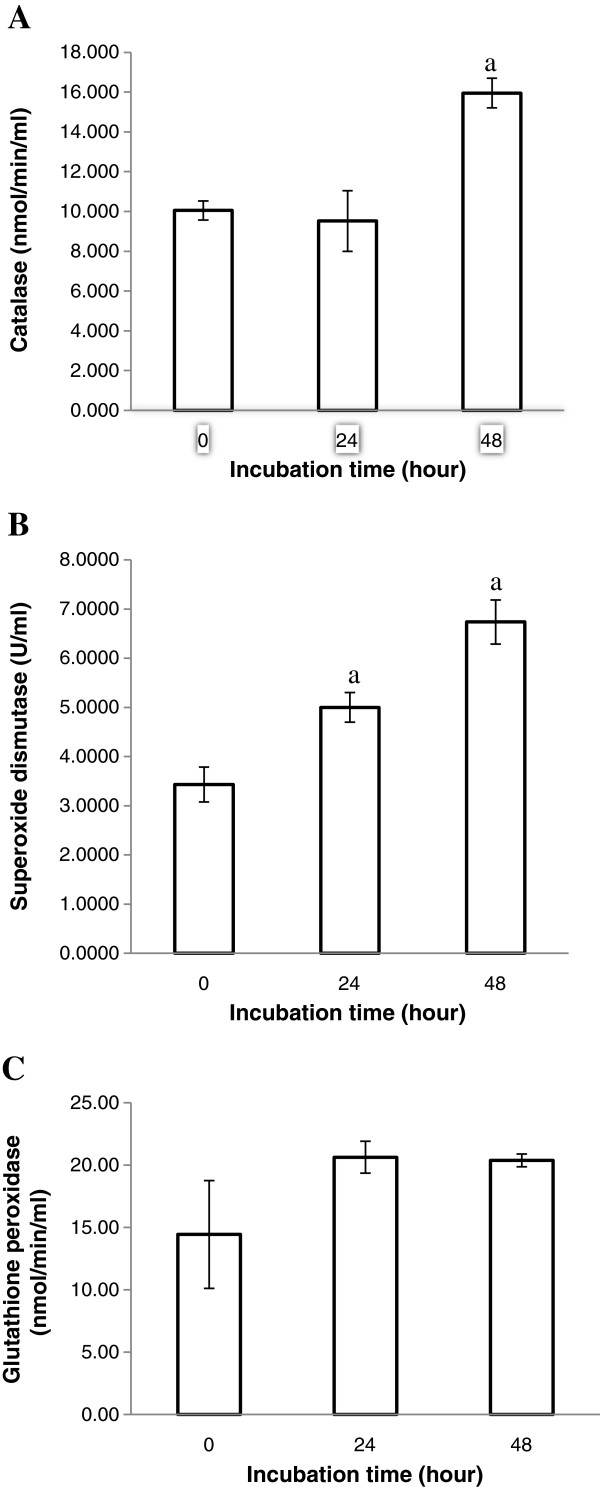
**(a-c) Activities of antioxidant enzymes in the MCF-7 cells before and after treatment with the ethyl acetate extracts of *P. betle. *** MCF-7 cells (1 x 10^6^) were treated with the IC_50_ concentration (65 μg/ml) of the ethyl acetate extract of *P. betle* for 0, 24 and 48 h. Activity of catalase (Figure 3a), superoxide dismutase (Figure 3b) and glutathione peroxidase (Figure 3c) was determined using commercial assay kits. a indicates significant difference from untreated cells (p<0.05).

The MCF-7 cells were treated with the IC_50_ concentration of the ethyl acetate extracts for 24 and 48 h and results are shown in Figure 3a. There was no difference in CAT activities between untreated and treated cells at 24 h (p>0.05), however CAT activities increased approximately 1.6 fold from control (p<0.05) at the 48 h incubation period.

### Superoxide dismutase activity

The ethyl acetate extracts of *P. betle* (64 μg/ml) was incubated with the MCF-7 cells for 24 and 48 h for determination of SOD activities. MCF-7 cells treated with the plant extract showed a time-dependent increase in SOD activities, almost doubling at the 48 h incubation point compared to untreated cells (Figure 3b).

### Glutathione peroxidase activity

The GPx activities of the treated cells increased slightly from 14.43 nmol/min/ml in the untreated cells to 20.63 nmol/min/ml and 20.38 nmol/min/ml at the 24 and 48 h incubation times, respectively (Figure 3c). However, these increases were not significantly different from the untreated cells (p<0.05).

## Discussion

Studies are on-going to search for natural-based anti-proliferative and chemopreventive agents which can act as alternatives to the chemically-synthesised drugs and which are potentially less toxic and contain less side effects. In this study, we tested the antioxidant abilities and cytotoxic effects of the extracts of *P. betle* on the breast cancer cells, MCF-7.

The antioxidant activities of *P. betle* have been reported in numerous studies but mostly concentrated on the aqueous or polar extracts. However, variation in antioxidant activities can still occur depending on varieties, location and growth conditions of the plant, hence data on antioxidant activities are still relevant and important [23,24]. In this study, we used solvents of varying polarities to separate antioxidants of low, medium and high polarity, using water, methanol, ethyl acetate and hexane, to provide a better insight into the antioxidative properties of this plant.

Overall, in the assessment of the antioxidant capacities of the plant extracts, the ethyl acetate extract showed the highest ferric reducing and radical scavenging activities against DPPH, superoxide anion and nitric oxide radicals. However, the ethyl acetate extract was not as potent as the aqueous extract in scavenging the hydroxyl radicals, implying selective scavenging effect of antioxidants in the former. Ethyl acetate is the most optimal solvent for extraction of antioxidants in *P. betle*, implying that the antioxidants in *P. betle* are mainly of medium polarity. In contrast, the antioxidant activities of the aqueous, methanol and hexane extracts were many folds lower than the ethyl acetate extract, implying minimal contribution of these extracts towards protection against oxidative damage. Many studies have reported positive correlation between phenolic compounds in plants and their antioxidant activities, showing the importance of phenolic compounds as antioxidants [25,26]. Our correlation analyses also support this observation. Indeed, the ethyl acetate extract of *P. betle* had the highest phenolic content, implying that the antioxidant activities may have been contributed by the phenolics. We identified the presence of catechin, morin and quercetin through HPLC analyses. These three compounds are well-known antioxidants and could have contributed to the observed antioxidant activities [27]. Previous studies have identified several phenolic compounds in the leaves of *P. betle* including β-sitosterol, dotriacontanoic acid, tritriacontane, stearic acid, hydroxychavicol, chevibetol and allylpyrocatechol, together with their glucosides [28,29]. Many of these compounds including chevibetol, hydroxychavicol and allylpyrocatechol have antioxidant activities [30,31].

Studies comparing the anti-proliferative effect of extracts of *P. betle* leaves on breast cancer cells are lacking. In this study, amongst the four solvent extracts, the ethyl acetate extract had the most potent anti-proliferative effect on breast cancer cells and this was observed to be more potent than *Scutellaria baicalensis*[32] and *Patrinia scabiosaefolia*[33]*,* popular Chinese medicinal herbs that are traditionally used for treating cancer. Furthermore, the ethyl acetate extract also contained the highest phenolic content and antioxidant activities which could contribute towards the protective effects. A study in China reported a positive correlation between the antioxidant activities of several Chinese medicinal herbs and their anti-cancer effects on MCF-7 cells [3]. Hydroxychavicol, a component of *P. betle* leaf showed anti-proliferative effect towards oral carcinoma cell line [30] and may have the same anti-proliferative effect against MCF-7 cells. Toxicology studies in rats showed no signs of toxicity and hepatotoxicity of *P. betle* up to a concentration of 1.5 g/kg body weight, implying its safety against normal cells and its specificity in targetting cancer cells [8].

Epidemiology and clinical studies have revealed the involvement of reactive oxygen species (ROS) in carcinogenesis [34,35]. Tumor cells have increased production of ROS, causing oxidative stress and disturbing the redox state, leading to DNA damage, mutations and altered gene expression which contributes to carcinogenesis. At the same time, cancer cells have reduced capacity to remove ROS due to altered antioxidant defense systems. However, ROS also play important roles in inducing apoptosis, implying an anti-cancer effect. Hence finding the right balance between ROS and antioxidant defense levels in cancer cells is important to ensure that cancer progression can be inhibited while at the same time maintaining apoptosis.

For this reason, we investigated the effect of the ethyl acetate extract on antioxidant enzyme levels in MCF-7-treated cells to ascertain the possible protective effects of these enzymes against oxidative damage. SOD catalyses the dismutation of superoxide anion into water and H_2_O_2_ whereas CAT and GPx protect against oxidative damage by converting H_2_O_2_ into water. Accumulation of H_2_O_2_ can lead to production of the highly reactive hydroxyl radicals, causing DNA damage.

CAT activation is postulated to exert control on breast cancer progression [36]. Indeed, activities of CAT have been reported to be lowered in breast cancer patients [37,38]. Acatalasemic and hypocatalasemic mice, which have drastically decreased CAT levels in the blood and tissues, were more susceptible to mammary carcinoma than their wild type counterparts [39]. A recent study reported that CAT overexpression in MCF-7 cells led to less proliferation and migration of the cancer cells [40]. The plant extract used in this study could have increased CAT activities possibly by directly inducing increased expression of CAT, thereby inhibiting proliferation of the cancer cells.

SOD activities are low in many cancers implying reduced protection against ROS [41,42]. Low levels of manganese superoxide dismutase (MnSOD) in nonaggressive breast cancer cells caused accumulation of superoxide anion which acted as second messengers, promoting cancer cell proliferation [43]. In contrast, over-expression of SOD in cancer cell lines, including MCF-7, inhibited tumor growth, possibly acting as tumor-supressor proteins [44]. One of the mechanisms for this is through the influence of MnSOD on transcription factor activity. Over-expression of MnSOD in MCF-7 cells have been reported to reduce transcriptional activity of the transcription factors AP-1 and NF-κB and decreased expression of interleukin IL-1 and IL-6 [45], contributing towards tumor supression. MnSOD can also supress tumors by altering ROS levels in cancer cells and it was reported that H_2_O_2_ production by MnSOD contributed to the tumor-supressing properties [43]. The increased SOD activities in our study imply the ability of the plant extract to remove superoxide anions and possibly inhibit tumor growth. Furthermore, the plant extract may also directly scavenge superoxide anion since the *in vitro* analysis showed its potent superoxide anion scavenging activity.

There are five different forms of GPx in humans, however, GPx1 and 4 are more relevant to breast cancer. Generally, all GPx could inhibit initiation and metastastis although this may differ according to the types and stages of the cancers [46]. GPx1 is postulated to prevent initiation of cancer through ROS-mediated DNA damage whereas GPx4 could inhibit growth of established tumors [47]. Our study did not show significant changes in GPx activities throughout the incubation times indicating the plant extract has little or no effect on this enzyme.

Antioxidant-rich plants such as raspberry, blueberry and soybeans could inhibit the growth of several cancer cell lines, including breast cancer [48]. Phenolic compounds such as epigallocatechin gallate, catechin, genistein and quercetin suppressed growth of breast cancer cells [1,2] implying the importance of antioxidants towards the anti-proliferative effects of cells. Anti-cancer agents with antioxidant activities may exert their beneficial effects by balancing levels of ROS so as not to cause further proliferation of cancer cells while still allowing apoptosis to occur. In addition to the radical scavenging properties of the ethyl acetate extract observed in this study, another study had also reported the ability of extracts of *P. betle* to scavenge ROS including H_2_O_2_, superoxide radicals and hydroxyl radicals [49] and this effect was attributed to hydroxychavicol, a major phenolic present in the plant [30]. Increased activities of the antioxidant enzymes in this study implied the ability of the extracts of *P. betle* to remove ROS and protect against oxidative damage while at the same time inhibiting cell proliferation. Studies have indicated that in addition to influencing antioxidant enzymes, antioxidants may inhibit carcinogenesis through other non-antioxidant action such as by modulating signaling pathways involved in cellular functions such as proliferation, cell growth and differentiation, by influencing activities of cancer-related enzymes such as cyclooxygenase-2 and phase I or II metabolizing enzymes or by inducing cell cycle arrest [50].

## Conclusion

In summary, the leaves of *P. betle* extracted with ethyl acetate contained the highest antoxidant activities and anti-proliferative effects against MCF-7 cells. We postulated that one of the possible action for the anti-proliferative effects of this extract occured through increased activities of antioxidant enzymes which helped in maintaining the balance between ROS production and removal. There is a great potential to develop *P. betle* as chemotherapeutic agents in breast cancer treatment, hence further studies are needed, particularly *in vivo* studies to further evaluate this effect.

## Competing interests

The authors declare that they have no competing interests.

## Authors’ contributions

NNA performed all the experiments and analysed the data. MSK designed the cytotoxicity study, supervised the experimental work and reviewed the final manuscript before submission. AAA designed the overall study, supervised the experimental work and wrote the manuscript. All authors read and approved the final manuscript.

## Pre-publication history

The pre-publication history for this paper can be accessed here:

http://www.biomedcentral.com/1472-6882/12/220/prepub
